# Comparison of short-term outcomes between SuperPATH approach and conventional approaches in hip replacement: a systematic review and meta-analysis of randomized controlled trials

**DOI:** 10.1186/s13018-020-01884-3

**Published:** 2020-09-17

**Authors:** Nikolai Ramadanov, Simon Bueschges, Kuiliang Liu, Roman Klein, Ruediger Schultka

**Affiliations:** 1 Center for Emergency Medicine, University Hospital Jena, Friedrich Schiller University, Am Klinikum 1, 07747 Jena, Germany; 2 Faculty of Medicine, Department of Statistics, University of Salamanca, Calle Espejo 2, 37007 Salamanca, Spain; 3 Department for Orthopaedics and Trauma Surgery, Siloah St. Trudpert Hospital, Wilferdinger Str. 67, 75179 Pforzheim, Germany; 4 Department for Orthopaedics, Trauma Surgery and Sports Traumatology, Marienhaus Hospital Hetzelstift, Stiftstr. 10, 67434 Neustadt, Germany; 5 Center for Surgery, Evangelical Hospital Ludwigsfelde-Teltow, Albert-Schweizer-Str. 40-44, 14974 Ludwigsfelde, Germany

## Abstract

**Background:**

It remains uncertain if the new SuperPATH approach benefits patients in artificial hip joint replacement. We conducted a systematic review and meta-analysis of randomized controlled trials to compare the short-term outcome of SuperPATH approach and conventional approaches in hip joint replacement.

**Methods:**

A systematic literature search up to April 2020 was performed to identify randomized controlled trials comparing SuperPATH with conventional approaches in hip joint replacement. We measured surgical, functional, and radiological outcomes. Mean differences or odds ratios with 95% confidence intervals were calculated and pooled using random effects models and the Hartung-Knapp-Sidik-Jonkman method.

**Results:**

A total of 12 RCTs involving 726 patients met the inclusion criteria, one trial with a level I evidence, 11 trials with level II evidence. The overall meta-analysis showed that SuperPATH approach reduced incision length (MD = − 4.84, 95% CI − 7.04 to − 2.64, *p* < 0.01), pain VAS 7 day postoperatively (MD = − 1.39, 95% CI − 2.57 to − 0.21, *p* = 0.03), and HHS 7 day postoperatively (MD = 10.24, 95% CI 0.27 to 20.21, *p* = 0.05). The two approaches did not differ in acetabular cup positioning angles, intra- and postoperative blood loss, hospitalization period, and postoperative complications. Hip replacement via SuperPATH approach had a longer operation time than hip replacement via conventional approaches.

**Conclusions:**

SuperPATH approach showed better results in decreasing incision length and early pain intensity as well as improvement of short-term functional outcome. Long-term outcomes of SuperPATH approach need to be investigated.

## Introduction

With the world population aging, the number of hip joint pathologies increases [1, 2]. Artificial hip replacement is one of the most effective treatments for many hip conditions. Approximately half of the hip fractures are femoral neck fractures. Numerous studies on the outcome of femoral neck fractures, operated by a head-preserving method, showed a high risk for osteosynthesis failure, in some cases > 40% [3–8]. In elderly patients with femoral neck fractures, artificial hip replacement should be considered [8].

Artificial total hip arthroplasty (THA) is applied since the 1920s. THA can improve pain, motor function of the hip joint, quality of life of the patient, and correct deformities [9]. According to a systematic review and meta-analysis, the survival at 15 years of THA is estimated with almost 90% [10]. Since long-term outcomes improved, there is still room for improvement in early postoperative recovery. Minimally invasive THA was developed to provide improvement [11]. Several approaches to the hip joint have been described and modified by various authors. An overview of conventional approaches is given in Table 1. However, conventional approaches damage muscles or tendons and remove the joint capsule, impairing stability and increasing the likelihood of postoperative dislocations [12]. Minimally invasive approaches are modifications of the conventional approaches. There is no uniform definition for minimally invasive approaches in hip replacement surgery. Some authors define an incision length of less than 10 cm as minimally invasive [13–15]. Others see the definition in less traumatization of the tissue [16–19]. The minimally invasive approaches are divided into two groups: “muscle-sparing” and “mini-incision” approaches. Over the last decades, several minimally invasive approaches and techniques have been introduced [20–30]. In general, there is no consensus in literature regarding a superiority of minimally invasive approaches compared to conventional approaches [31–38].

**Table 1:** Overview of conventional approaches to the hip joint in hip replacement

Conventional approaches	Described by
Anterior approach	Carl Hueter (1881), Smith-Petersen (1949), Judet (1985)
Anterolateral approach	Sayre (1884), Watson-Jones (1936)
Lateral transgluteal approach	Bauer (1979), Hardinge (1982)
Lateral transtrochanteric approach	Charnley (1970)
Posterior approach	Langenbeck (1874), Kocher (1902), Gibson (1950)
Posterolateral approach	Marcy and Fletcher (1954)

A relatively new and promising minimally invasive approach to the hip joint for hip replacement is the supercapsular percutaneously assisted approach in total hip arthroplasty (SuperPATH). The SuperPATH approach was introduced and reported by James Chow in 2011 [39]. It is described as follows: the incision of the capsule is performed through a 6–10-cm skin incision and a muscle-sparing approach between the piriformis and gluteus minimus muscles in lateral decubitus position. The femoral canal is then opened with a reamer. Thereafter, the femur is broached and osteotomy of the femoral neck is performed. After preparation of the acetabulum, the acetabular basket reamer is connected through the main incision with the drilling machine through a percutaneous portal. After implantation of the cup, inlay, modular neck and head, reposition is performed. Conventional wound closure concludes the operation.

Since its introduction, several studies were conducted to reveal differences in outcomes of SuperPATH approach in comparison to conventional approaches in hip replacement. The conclusions of these studies are varying [40–58]. While English literature on this subject appears sparse [40–42], most of the studies are published in Chinese [43–57]. There is one noteworthy study in Spanish [58]. Furthermore, there are two Chinese systematic reviews and meta-analyses, comparing the outcomes between SuperPATH and conventional approaches in hip replacement [59, 60]. Unfortunately, one of them only contains four trials, one of which being an observational study [60]. The other Chinese meta-analysis stated to have included eight randomized controlled trials (RCTs) [59]. Two of them claim to be retrospective, another one to be prospective in Chinese, but retrospective in the abstract translated to English [54, 55, 57]. Nevertheless, this study is not categorized as “randomized” [55]. In addition, the confounding of hemiarthroplasty (HA) [48] and THA as well as conventional and mini-incision approaches [56] poses another severe limitation to this meta-analysis [59].

The aim of this systematic review and meta-analysis was to compare the short-term outcome of SuperPATH minimally invasive approach and conventional approaches in hip replacement for treatment of hip joint diseases and fractures, including only quality RCTs.

## Methods

### Reporting guidelines and protocol registration

We followed the Preferred Reporting Items for Systematic Reviews and Meta-Analysis-Protocols (PRISMA-P) guidelines [61]. The review protocol was registered with the International Prospective Register of Systematic Reviews (PROSPERO) on 22 March 2020 and finally approved on 28 April 2020 (CRD42020175859) at http://www.crd.york.ac.uk/PROSPERO/

### Data sources and search strategies

We searched the following databases and checked citations of screened studies and reviews for relevant manuscripts.

- PubMed
- Chinese National Knowledge Infrastructure (CNKI)
- The Cochrane Library
- Google Scholar
- Clinical Trials

We built a BOOLEAN search strategy (see appendix) and adapted it to the syntax of the used databases. No restrictions to publication date or language apply. Results of the searches were exported to a reference management software [62]. A Chinese-speaking reviewer (KL) helped with the search in CNKI.

### Study screening and selection

Two independent reviewers (NR and RS) scanned titles and abstracts to select articles for further consideration. The full text of the selected articles was obtained and scanned again for inclusion by the two reviewers (NR and RS). The decision on inclusion of each study was determined by the consensus between the two reviewers. Cases of disagreement were resolved by discussion and consensus with a third reviewer (RK). Kappa coefficient was used to measure the agreement between the reviewers. A Chinese-speaking reviewer (KL) helped with translation aspects of the study screening and selection.

### Inclusion criteria

Types of studies are as follows:

- Randomized controlled trials

Types of participants are as follows:

- Human participants with hip disease or hip fracture

Types of interventions are as follows:

- THA and HA via SuperPATH and conventional approaches

### Exclusion criteria

- No outcome of interest
- Mini-incision approaches
- Employment of a computer navigation system

### Types of outcome measures

1. Surgical outcome
  - The operation time (in minutes) was defined as period of time from the beginning of skin incision to suture. It correlates with the competence of the surgeon in these two different approaches as well as risk of infection.
  - The incision length (in centimeters) was measured on graduated scale. It reflects the severity intraoperative trauma.
  - The intraoperative blood loss (in milliliters) was defined as the total amount of blood from the suction device. It reflects the severity of intraoperative trauma.
  - The postoperative drainage volume (in milliliters) was defined as the total amount of blood collected in the drainage bag.
  - The pain visual analog scale (VAS) is an instrument for measuring pain intensity, providing a range of scores from 0 to 10 [63, 64]. The degree of hip pain was evaluated at periodically time intervals after operation.
  - The hospitalization period (in days) was the time period from admission to discharge of the patient.
2. Functional outcome
  - The Harris Hip Score (HHS) was developed for assessment of the results of hip surgery [65]. The hip joint function was periodically evaluated at time intervals after operation. The score collects points from the assessment of four aspects: pain, function, degree of deformity, and range of motion of the hip. The higher the added score, the better the results, providing a range of added scores from 0 to 100.
3. Radiological outcome
  - The acetabular cup anteversion angle and (b) the inclination angle (in degrees) have ideal values for positioning: anteversion angle from 10 to 25° and inclination angle from 40 to 50° [66]. Especially, the ideal acetabular cup anteversion is of great importance, since an angle too big often leads to anterior dislocation and an angle too small leads to posterior dislocation.
4. Postoperative complications such as venous thrombosis of lower extremities, implant loosening, infection, periprosthetic fracture, or dislocation were investigated.

### Data extraction and analysis

Data extraction was performed by two reviewers (NR and RS). Cases of disagreement were resolved by discussion and consensus with a third reviewer (RK). We extracted all relevant data into a data extraction form in a standard electronic spreadsheet and the Cochrane software program Review Manager Version 5.3 [67]. We extracted the following data: first author, year of publication, number of patients, patient characteristics, study design, risk of bias, and outcome. A Chinese-speaking reviewer (KL) helped with the language-dependent aspects of data extraction and analysis.

### Assessment for risk of bias and level of evidence

We examined and checked the selected studies for their risk of bias. We made an assessment using Cochrane’s Risk of Bias 2 (RoB 2) tool [68]. The level of evidence was rated for each study, in accordance with guidelines of the Centre for Evidence-Based Medicine (Oxford, UK) [69].

### Statistical analysis

#### Measures of treatment effect

In statistical calculations the SuperPATH approach group was “experimental group” and the conventional approach group was the “control group”. We calculated the odds ratio (OR) and their 95% confidence intervals (CIs) for dichotomous outcomes. An odds ratio of less than 1 favored the experimental group. We calculated mean differences (MDs) with 95% CIs for continuous outcomes. Furthermore, we calculated prediction intervals to estimate where to expect the next data point sampled. We calculated the *t* test to determine statistically significant differences between the means of the two groups. We used a significance level of *p* = 0.05. We evaluated the results and analyzed them on basis of the Cochrane Handbook for Systematic Reviews of Interventions [70], Cochrane’s Review Manager Version 5.3 [67], and the R packages meta [71] and metafor [72]. In case of relevant clinical heterogeneity attributable to the individual studies’ PICOs we did not pool data and reported a narrative review. Otherwise the pooled effect sizes, as well as 95% confidence intervals [CIs], were calculated with both the Hartung-Knapp-Sidik-Jonkman method and the DerSimonian and Laird method, using both fixed and random effects models to estimate the variance of the distribution of true effect sizes. The Hartung-Knapp-Sidik-Jonkman method gave the most conservative confidence intervals. Therefore, it was chosen for presenting the results in accordance with current literature [73, 74]. Study weighting was performed by inverse variance or Mantel-Haenszel method [74, 75].

#### Assessment of heterogeneity

We assessed clinical and statistical heterogeneity. We did not pool study data that were clinically too diverse. Heterogeneity was assessed using Cochrane’s *Q* test (*p* value < 0.10 is indicative of heterogeneity) and Higgins’ test *I*^2^ (low heterogeneity, < 25%; moderate heterogeneity, 25–75%; and high heterogeneity, > 75%) [76].

#### THA-subgroup analysis

In addition to pooling the effect sizes of all studies, we compared the model results for RCTs with a THA only and additionally reported their results if they differed from the overall effect in a clinically relevant manner.

## Results

### Study identification and selection

**Fig. 1:**
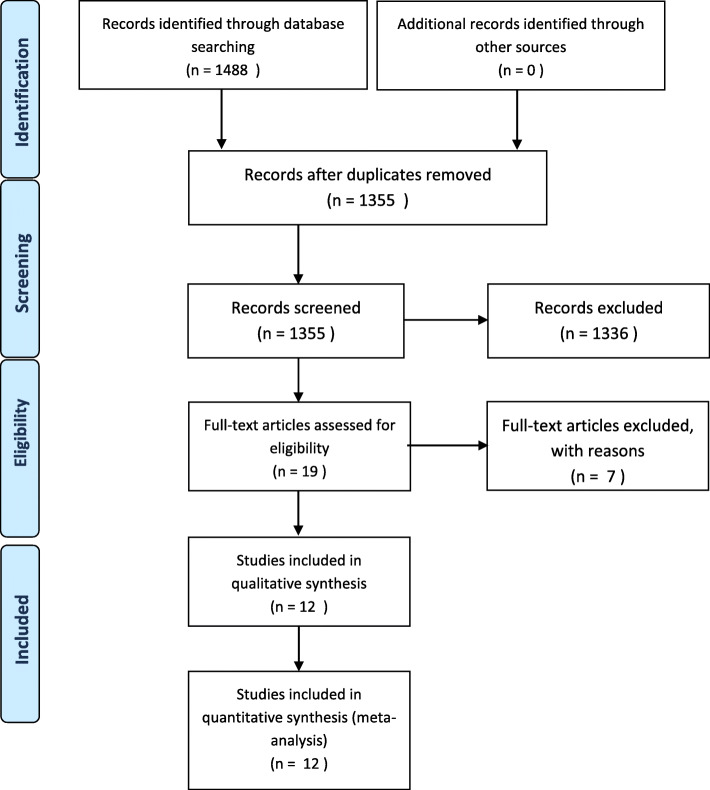
PRISMA flow diagram of the search results and selection according to our inclusion criteria

After removing 133 duplicates, a total of 1355 studies were identified in our initial literature search. Nineteen studies were assessed for eligibility after first screening procedure by title and abstract (*κ* = 1.0) with total agreement by the reviewers. Of these studies, 7 were excluded after second screening procedure by full-paper analysis (*κ* = 1.0), leaving a total of 12 studies for inclusion in final meta-analysis. A description of the study selection process is given in a PRISMA flow diagram (see Fig. 1) [77].

### Characteristics of the RCTs

Table 2 shows the main characteristics of the 12 included RCTs. These studies were published between 2016 and 2020, altogether involving 726 patients (with 749 operated hip joints). Three hundred and forty-six of the included patients were operated via SuperPATH approach and 380 of the included patients were operated via conventional approaches. The sample size of these trials ranged from 4 to 154 patients. Two studies were published in English language [41, 42], 1 study was published in Chinese language only [45], and the other 9 studies were published in Chinese with an English abstract [43, 44, 46–52]. The main preoperative diagnoses were osteoarthritis, femoral neck fracture, and avascular necrosis of the femoral head. Of the 12 studies, 7 included hip replacements via the posterolateral approach [41, 43–45, 47, 48, 50], 1 via posterior approach [42], and 1 via lateral approach [51]. In 3 studies, the surgical approach was conventional, but not further specified.

**Table 2:** Main characteristics of RCTs included in meta-analysis

Study	Sample Size, n		THA/HA, n	Surgical approach		Mean Age, y (SD or range)		Gender (M/F), n		BMI, kg/m^2^ (SD or range)		Preoperative hip pathology					
				SG	pl, p, l, c							OA		Fx		ANFH	
	Pts	Hips			CG	SG	CG	SG	CG	SG	CG	SG	CG	SG	CG	SG	CG
Meng et al. [41]	4	8	THA	4	4 pl	51.00 ± 4.54		4/0		21.49 (19.60–23.04)		0		0		8	
Ouyang et al. [43]	24	24	THA	12	12 pl	54 (45–71)	55 (47–67)	8/4	9/3	23.1 ± 17.5–26.7	23.9 (16.9–30.4)	5	0	0	0	0	6
Yuan et al. [44]	84	84	THA	40	44 pl	74.3 (67–79)	75.7 (69–82)	24/16	21/23	22.73 ± 1.71	22.36 ± 1.89	5	6	21	24	10	12
Xie et al. [42]	92	92	THA	46	46 p	66.6 ± 11.88	64.47 ± 12.09	12/34	19/27	23.62 ± 1.63	24.06 ± 2.72	N/A					
Huang et al. [45]	38	38	THA/HA	18	20pl	76.6 ± 71–85	77.5 (70–80)	7/11	6/14	< 30	< 30	0		18	20	0	
Xu et al. [46]	92	92	HA	46	46 c	70.81 ± 6.0	71.02 ± 5.96	25/21	28/18	N/A	N/A	0		46	46	0	
Zhang et al. [47]	54	54	THA	27	27 pl	62.41 ± 6.44	61.28 ± 6.7	10/17	12/15	24.53 ± 5.31	23.93 ± 4.89	7	9	N/A		15	14
Wu et al. [48]	40	40	HA	20	20 pl	80.5 (75–89)		12/28		N/A	N/A	0		40		0	
Hou et al. [49]	40	40	THA	20	20 c	54.3 ± 13.7	53.8 ± 12.9	13/7	12/8	24.5 ± 3.6	23.9 ± 4.1	6	5	0		14	15
Xia et al. [50]	62	62	HA	30	32 pl	81 ± 4.57	80.66 ± 4.26	8/22	11/21	N/A	N/A	0		30	32	0	
Yan et al. [51]	154	173	THA	70	103 l	66 (59–75)	65 (56–82)	29/35	42/48	24.5 (17.3–31.1)	23.6 (18–32.3)	N/A		11	23	39	55
Ren et al. [52]	42	42	THA	21	21 c	57.96 ± 6.89	58.45 ± 6.25	12/9	13/8	N/A	N/A	0		0		21	21

*SG* SuperPATH approach group, *CG* conventional approach group, *THA* total hip arthroplasty, *HA* hemiarthroplasty, *pl* posterolateral approach, *p* posterior approach, *l* lateral approach, *c* conventional approach, *OA* osteoarthritis, *Fx* fracture, *ANFH* avascular necrosis of the femoral head, *Pts* patients

### Risk of bias and level of evidence

**Fig. 2:**
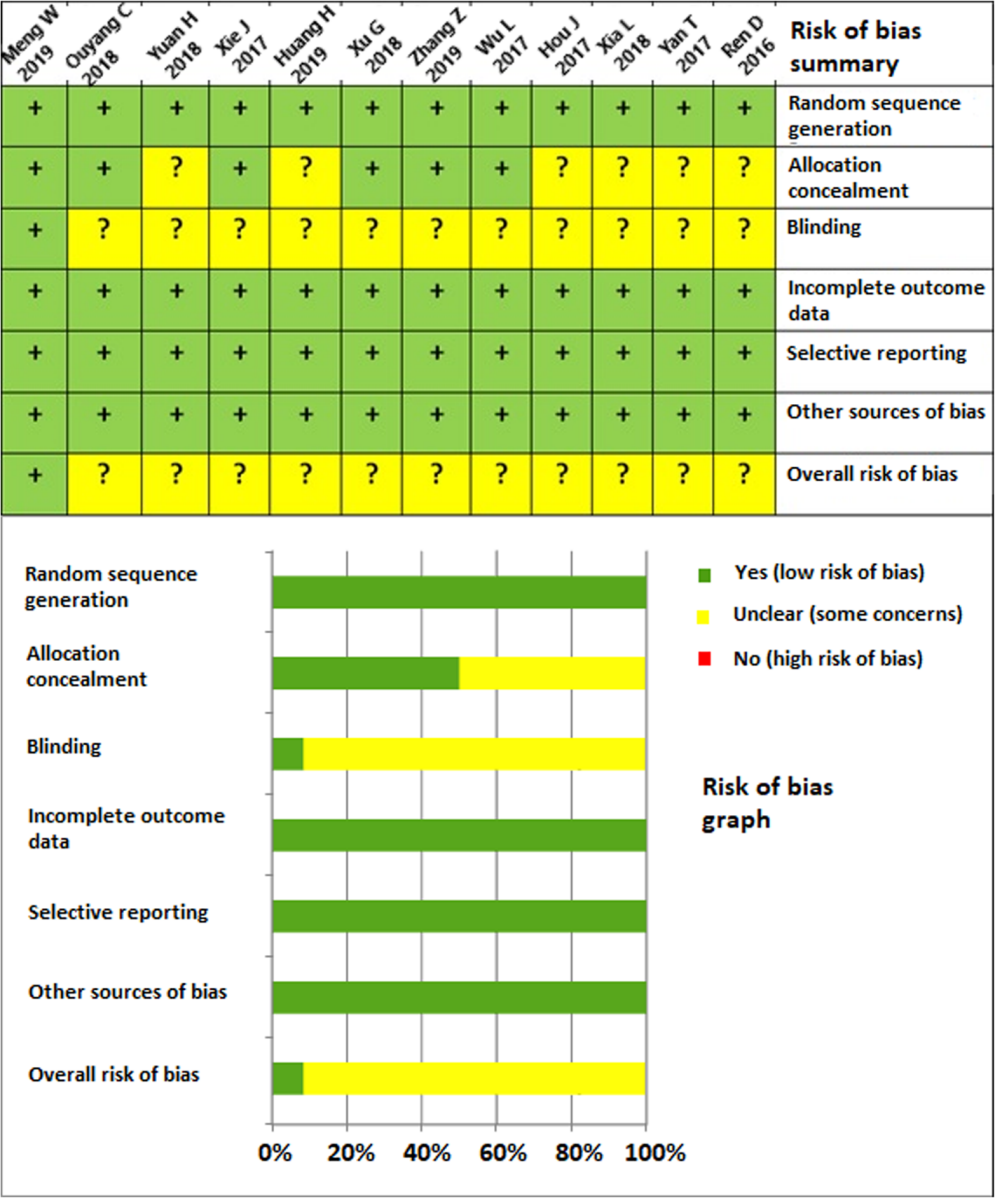
Risk of bias summary and graph

The quality of the included studies was assessed by the Cochrane Collaboration’s tool for risk of bias. Figure 2 shows the summarized assessment for risk of bias in a risk of bias summary and a risk of bias graph. One study out of twelve was a blinded RCT with a level I evidence [41], the other 11 studies were non-blinded RCTs with level II evidence [42–52].

### Clinical and statistical heterogeneity

**Fig. 3:**
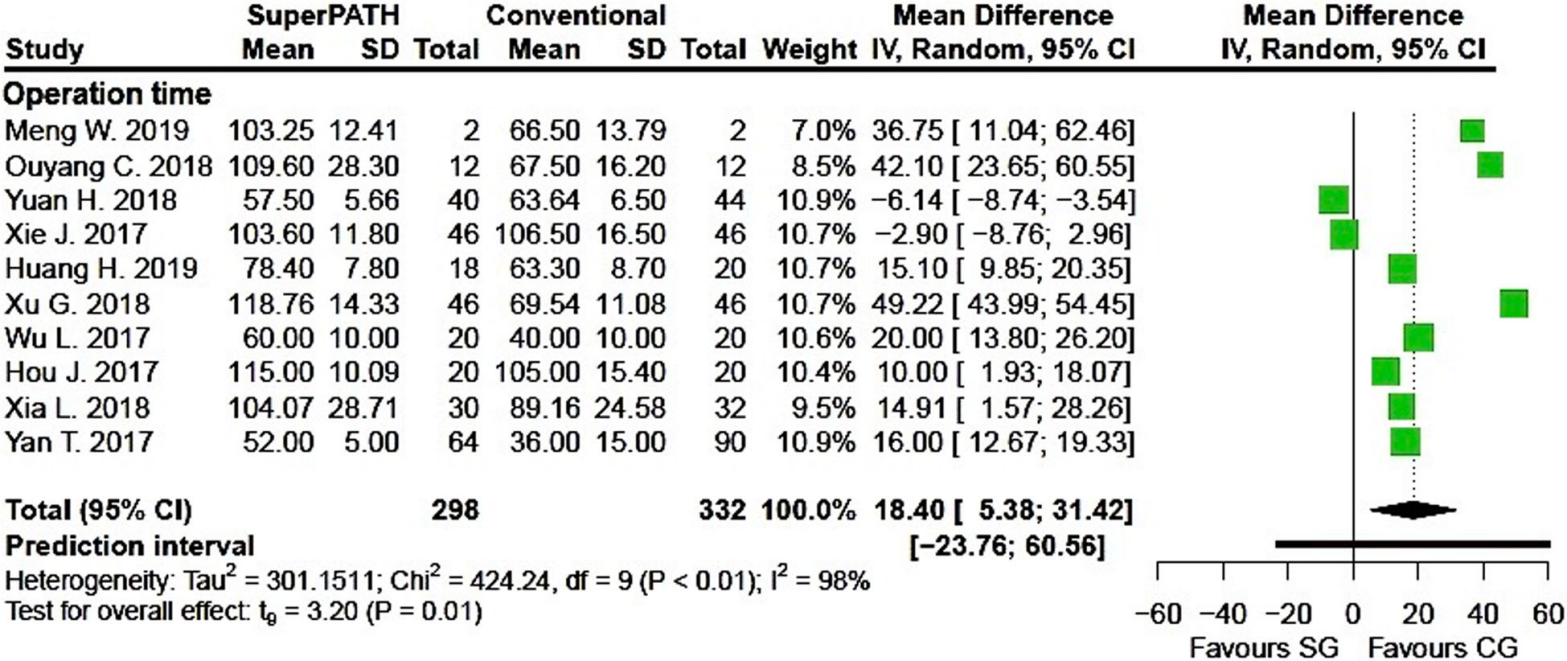
Comparison of the operation time in minutes in SuperPATH approach and conventional approach groups. SG, SuperPATH approach group; CG, conventional approach group; IV, inverse variance; CI, confidence interval

**Fig. 4:**
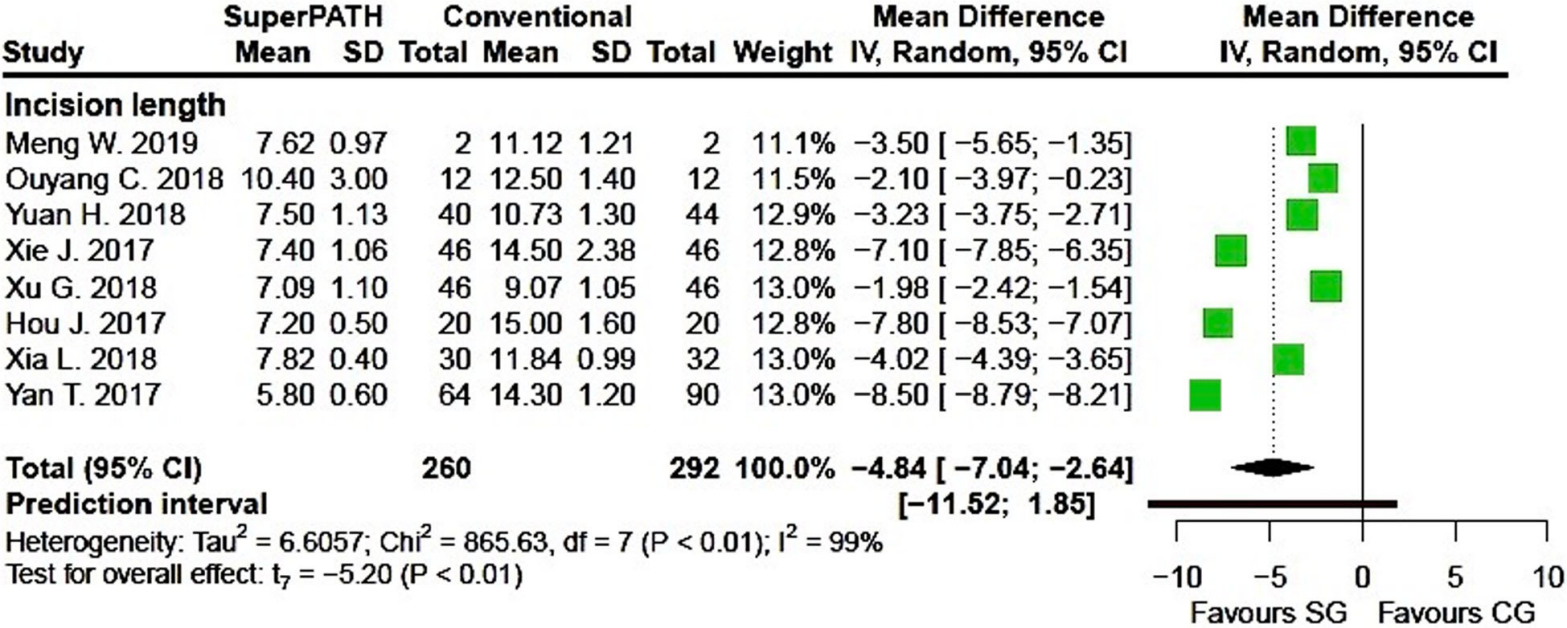
Comparison of the incision length in cm in SuperPATH approach and conventional approach groups. SG, SuperPATH approach group; CG, conventional approach group; IV, inverse variance; CI, confidence interval

**Fig. 5:**
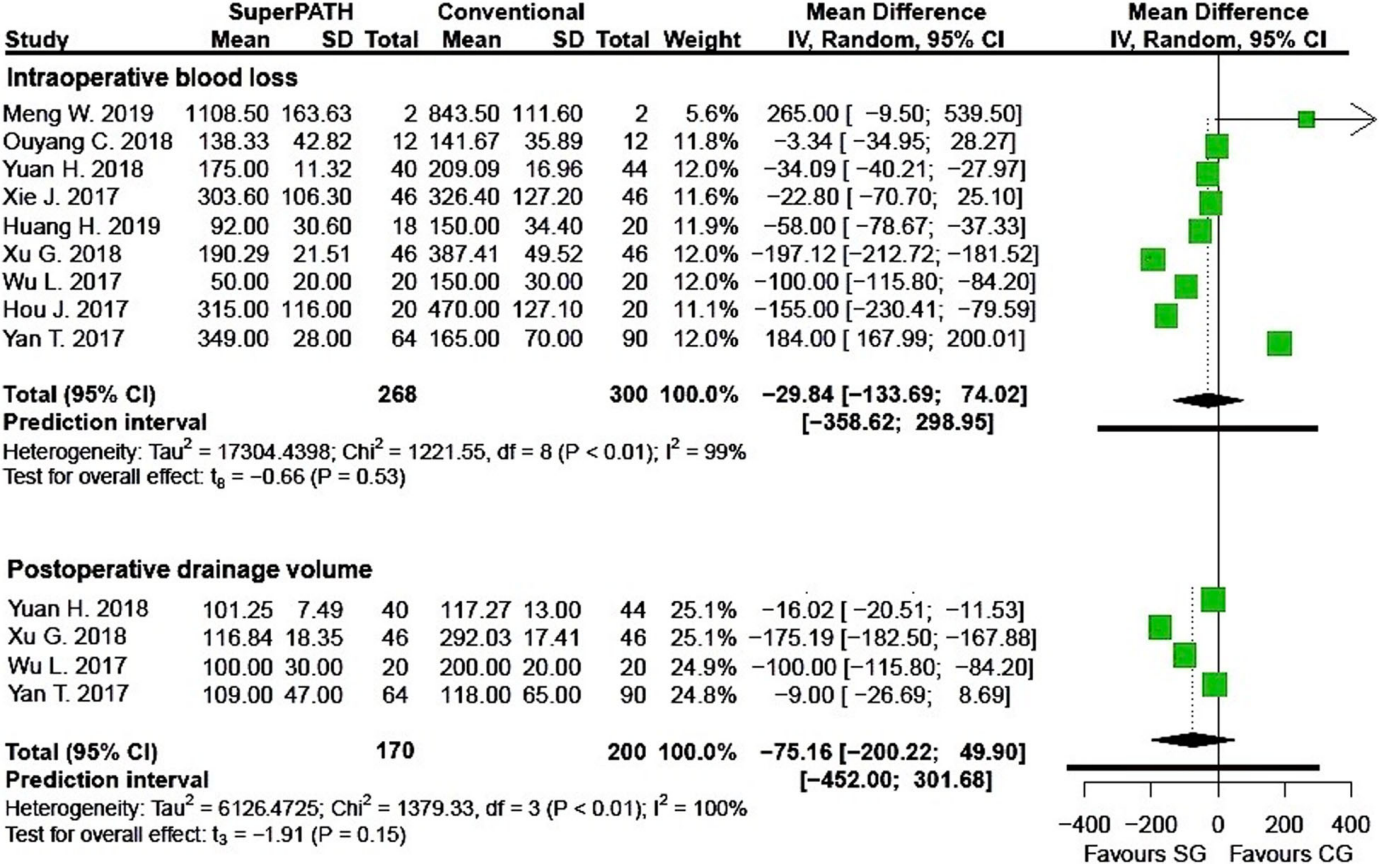
Comparison of the intraoperative blood loss and the postoperative drainage volume in ml in SuperPATH approach and conventional approach groups. SG, SuperPATH approach group; CG, conventional approach group; IV, inverse variance; CI, confidence interval

**Fig. 6:**
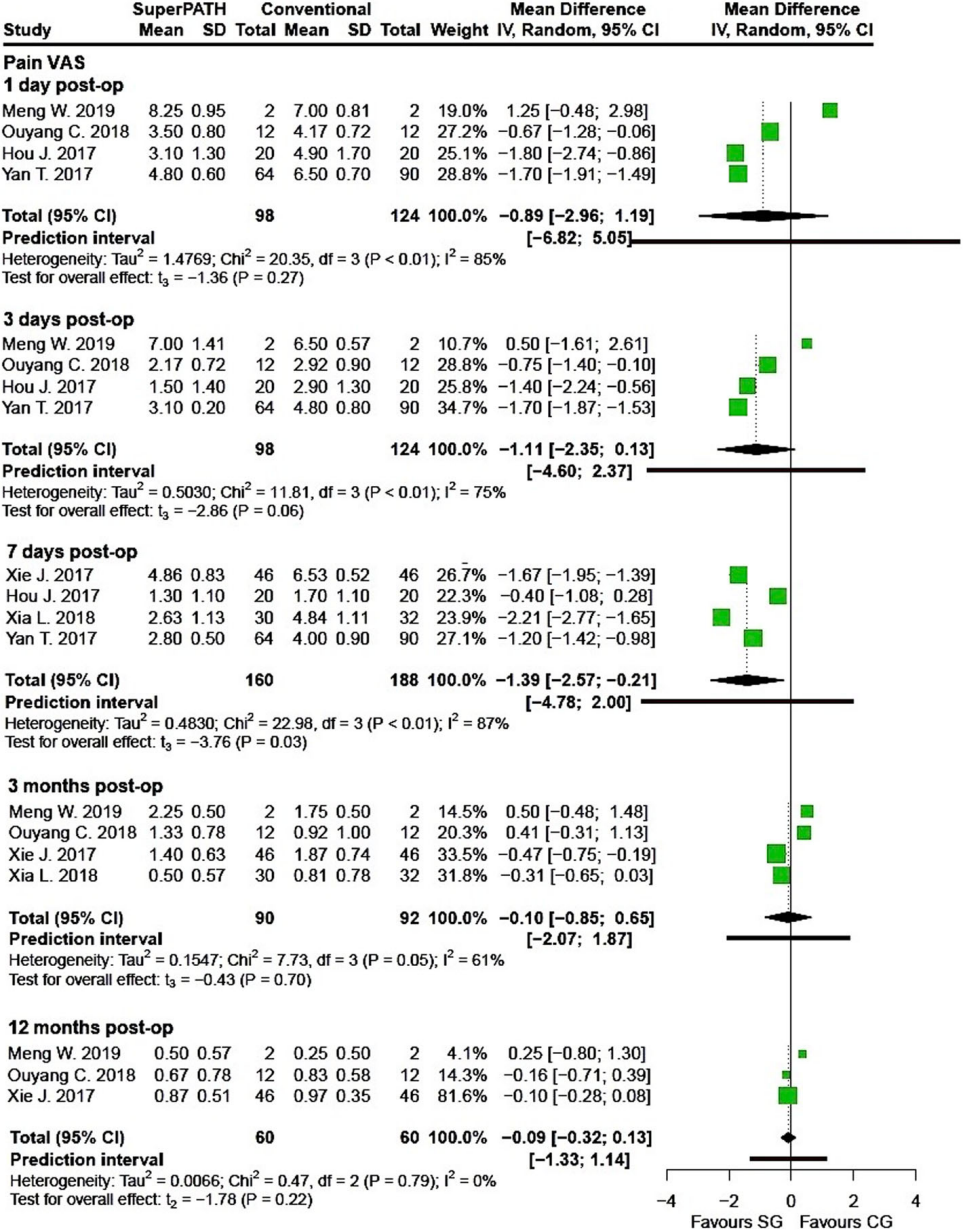
Comparison of the pain VAS 1, 3, 7 days, 3 and 12 months postoperatively in SuperPATH approach and conventional approach groups. SG, SuperPATH approach group; CG, conventional approach group; IV, inverse variance; CI, confidence interval; post-op, postoperative

**Fig. 7:**
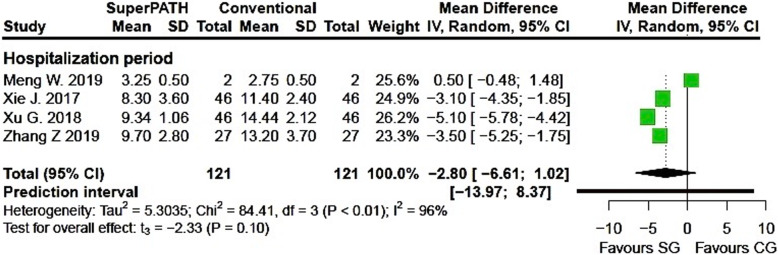
Comparison of the hospitalization period in days in SuperPATH approach and conventional approach groups. SG, SuperPATH approach group; CG, conventional approach group; IV, inverse variance; CI, confidence interval

**Fig. 8:**
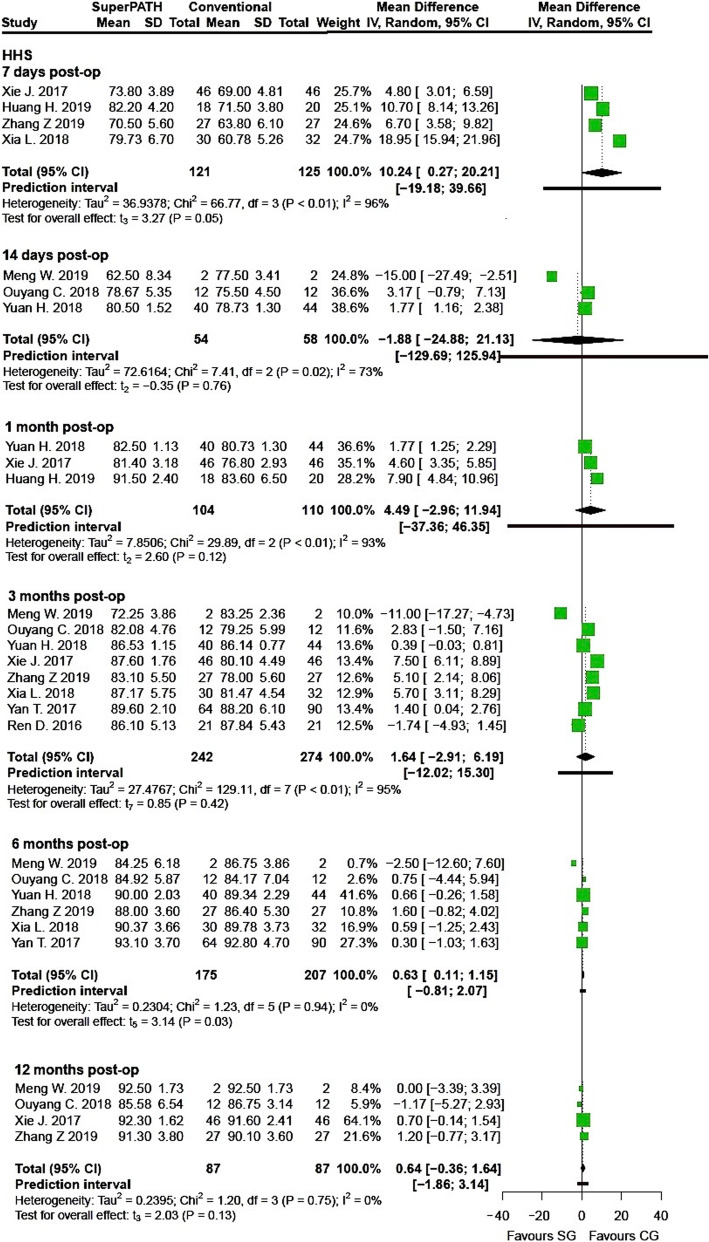
Comparison of the HHS 7, 14 days, 1, 3, 6 and 12 months postoperatively in SuperPATH approach and conventional approach groups. SG, SuperPATH approach group; CG, conventional approach group; IV, inverse variance; CI, confidence interval; HHS, Harris Hip Score; post-op, postoperatively

**Fig. 9:**
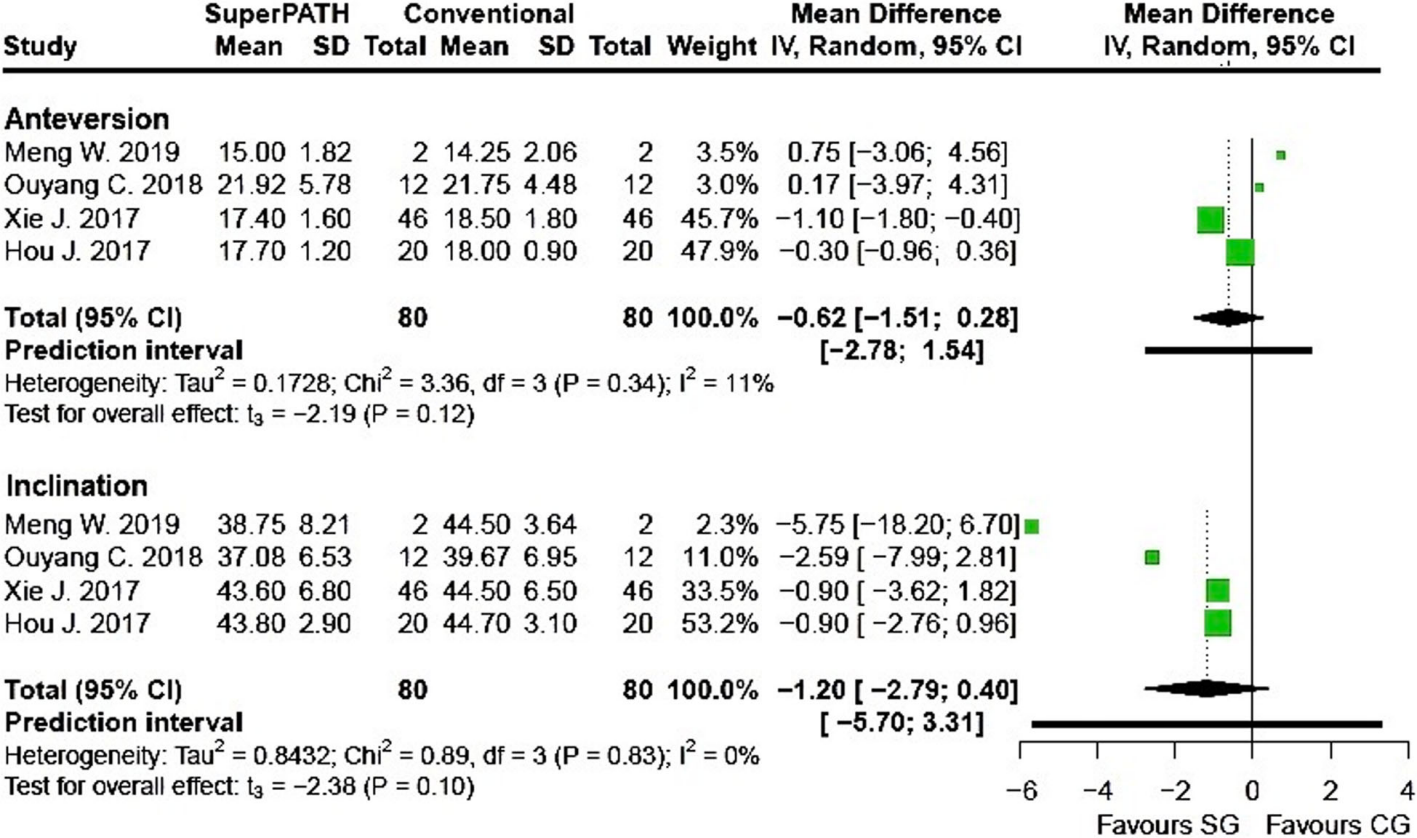
Comparison of the acetabular cup positioning angle in degrees in SuperPATH approach and conventional approach groups. SG, SuperPATH approach group; CG, conventional approach group; IV, inverse variance; CI, confidence interval

**Fig. 10:**
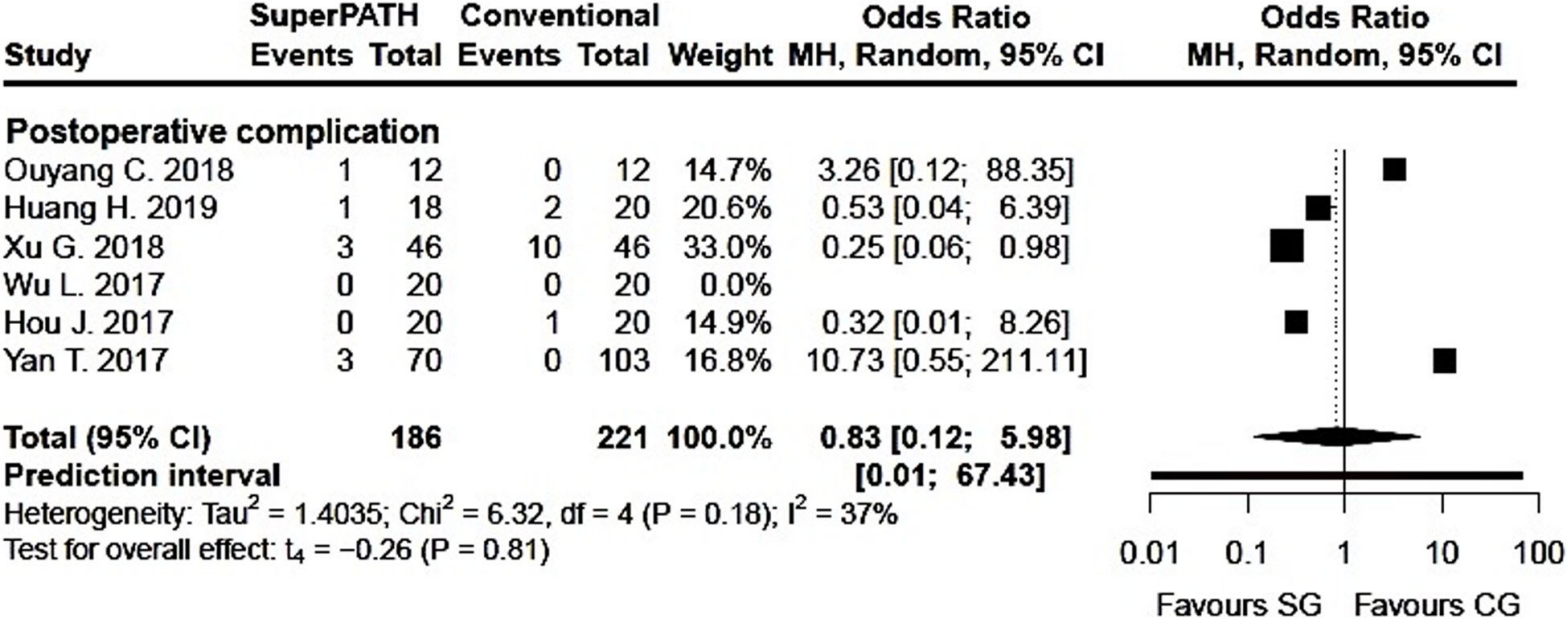
Comparison of postoperative complications in SuperPATH approach and conventional approach groups. SG, SuperPATH approach group; CG, conventional approach group; MH, Mantel-Haenszel; CI, confidence interval

Clinical characteristics for gender, age, and BMI (see Table 2) did not show relevant differences between the patients in the SuperPATH and conventional approach group. Ten out of 19 measured outcomes showed a high statistical heterogeneity (> 75%), four out of 19 measured outcomes showed a moderate statistical heterogeneity (25–75%), and 5 out of 19 measured outcomes showed a low statistical heterogeneity (< 25%) (see Figs. 3, 4, 5, 6, 7, 8, 9, and 10).

### Outcomes

#### 1. Surgical outcomes

##### Operation time

Data on 630 patients (including 298 patients with SuperPATH approach and 332 patients with conventional approaches) were pooled from 10 RTCs analyzing the operation time. Compared with the conventional approach group, the operation time of the SuperPATH group was 18.4 min longer (MD = 18.40, 95% CI 5.38 to 31.42, *I*^2^ = 98%, *p* = 0.01, Fig. 3).

##### Incision length

Data on 552 patients (including 260 patients with SuperPATH approach and 292 patients with conventional approaches) were pooled from 8 RTCs analyzing the incision length. Compared with the conventional approach group, the incision length of the SuperPATH group was 4.8 cm shorter (MD = − 4.84, 95% CI − 7.04 to − 2.64, *I*^2^ = 99%, *p* < 0.01, Fig. 4).

##### Intraoperative blood loss

Data on 568 patients (including 268 patients with SuperPATH approach and 300 patients with conventional approaches) were pooled from 9 RTCs analyzing the intraoperative blood loss. There was no difference between the conventional approach group and the SuperPATH group, concerning the intraoperative blood loss (MD = − 29.84, 95% CI − 133.69 to 74.02, *I*^2^ = 99%, *p* = 0.53, Fig. 5).

##### Postoperative drainage volume

Data on 370 patients (including 170 patients with SuperPATH approach and 200 patients with conventional approaches) were pooled from 4 RTCs analyzing the postoperative drainage volume. There was no difference between the conventional approach group and the SuperPATH group, concerning the postoperative drainage volume (MD = − 75.16, 95% CI − 200.22 to 49.90, *I*^2^ = 100%, *p* = 0.15, Fig. 5).

##### Pain visual analog scale

*Pain VAS 1 day postoperatively* Data on 222 patients (including 98 patients with SuperPATH approach and 124 patients with conventional approaches) were pooled from 4 RTCs analyzing the pain VAS 1 day postoperatively. There was no difference between the conventional approach group and the SuperPATH group, concerning the pain VAS 1 day postoperatively (MD = − 0.89, 95% CI − 2.96 to 1.19, *I*^2^ = 85%, *p* = 0.27, Fig. 6).

*Pain VAS 3 days postoperatively* Data on 222 patients (including 98 patients with SuperPATH approach and 124 patients with conventional approaches) were pooled from 4 RTCs analyzing the pain VAS 3 days postoperatively. There was no difference between the conventional approach group and the SuperPATH group, concerning the pain VAS 3 days postoperatively (MD = − 1.11, 95% CI − 2.35 to 0.13, *I*^2^ = 75%, *p* = 0.06, Fig. 6).

*Pain VAS 7 days postoperatively* Data on 348 patients (including 160 patients with SuperPATH approach and 188 patients with conventional approaches) were pooled from 4 RTCs analyzing the pain VAS 7 days postoperatively. Compared with conventional approach group, the pain VAS of the SuperPATH group was 1.4 points lower 7 days postoperatively (MD = − 1.39, 95% CI − 2.57 to − 0.21, *I*^2^ = 87%, *p* = 0.03, Fig. 6).

*Pain VAS 3 months postoperatively* Data on 182 patients (including 90 patients with SuperPATH approach and 92 patients with conventional approaches) were pooled from 4 RTCs analyzing the pain VAS 3 months postoperatively. There was no difference between the conventional approach group and the SuperPATH group, concerning the pain VAS 3 months postoperatively (MD = − 0.10, 95% CI − 0.85 to 0.65, *I*^2^ = 61%, *p* = 0.70, Fig. 6).

*Pain VAS 12 months postoperatively * Data on 120 patients (including 60 patients with SuperPATH approach and 60 patients with conventional approaches) were pooled from 3 RTCs analyzing the pain VAS 12 months postoperatively. There was no difference between the conventional approach group and the SuperPATH group, concerning the pain VAS 12 months postoperatively (MD = − 0.09, 95% CI − 0.32 to 0.13, *I*^2^ = 0%, *p* = 0.22, Fig. 6).

*Hospitalzation period* Data on 242 patients (including 121 patients with SuperPATH approach and 121 patients with conventional approaches) were pooled from 4 RTCs analyzing the hospitalization period. There was no difference between the conventional approach group and the SuperPATH group, concerning the hospitalization period (MD = − 2.80, 95% CI − 6.61 to 1.02, *I*^2^ = 96%, *p* = 0.10, Fig. 7).

#### 2. Functional outcome

##### Harris hip score

*HHS 7 days postoperatively* Data on 246 patients (including 121 patients with SuperPATH approach and 125 patients with conventional approaches) were pooled from 4 RTCs analyzing the HHS 7 days postoperatively. Compared with conventional approach group, the HHS of the SuperPATH group was 10.2 points higher 7 days postoperatively (MD = 10.24, 95% CI 0.27 to 20.21, *I*^2^ = 96%, *p* = 0.05, Fig. 8).

*HHS 14 days postoperatively* Data on 112 patients (including 54 patients with SuperPATH approach and 58 patients with conventional approaches) were pooled from 3 RTCs analyzing the HHS 14 days postoperatively. There was no difference between the conventional approach group and the SuperPATH group, concerning the HHS 14 days postoperatively (MD = − 1.88, 95% CI − 24.88 to 21.13, *I*^2^ = 73%, *p* = 0.76, Fig. 8).

*HHS 1 month postoperatively* Data on 214 patients (including 104 patients with SuperPATH approach and 110 patients with conventional approaches) were pooled from 3 RTCs analyzing the HHS 1 month postoperatively. There was no difference between the conventional approach group and the SuperPATH group, concerning the HHS 1 month postoperatively (MD = 4.49, 95% CI − 2.96 to 11.94, *I*^2^ = 93%, *p* = 0.12, Fig. 8).

*HHS 3 months postoperatively* Data on 516 patients (including 242 patients with SuperPATH approach and 274 patients with conventional approaches) were pooled from 8 RTCs analyzing the HHS 3 months postoperatively. There was no difference between the conventional approach group and the SuperPATH group, concerning the HHS 3 months postoperatively (MD = 1.64, 95% CI − 2.91 to 6.19, *I*^2^ = 95%, *p* = 0.42, Fig. 8).

*HHS 6 months postoperatively* Data on 382 patients (including 175 patients with SuperPATH approach and 207 patients with conventional approaches) were pooled from 6 RTCs analyzing the HHS 6 months postoperatively. Compared with conventional approach group, the HHS of the SuperPATH group was 0.6 points higher 6 months postoperatively (MD = 0.63, 95% CI 0.11 to 1.15, *I*^2^ = 0%, *p* = 0.03, Fig. 8).

*HHS 12 months postoperatively* Data on 174 patients (including 87 patients with SuperPATH approach and 87 patients with conventional approaches) were pooled from 4 RTCs analyzing the HHS 12 months postoperatively. There was no difference between the conventional approach group and the SuperPATH group, concerning the HHS 12 months postoperatively (MD = 0.64, 95% CI − 0.36 to 1.64, *I*^2^ = 0%, *p* = 0.13, Fig. 8).

#### 3. Radiological outcome

##### Acetabular cup anteversion angle

Data on 160 patients (including 80 patients with SuperPATH approach and 80 patients with conventional approaches) were pooled from 4 RTCs analyzing the cup anteversion angle. There was no difference between the conventional approach group and the SuperPATH group, concerning the cup anteversion angle (MD = − 0.62, 95% CI − 1.51 to 0.28, *I*^2^ = 11%, *p* = 0.12, Fig. 9).

##### Acetabular cup inclination angle

Data on 160 patients (including 80 patients with SuperPATH approach and 80 patients with conventional approaches) were pooled from 4 RTCs analyzing the cup inclination angle. There was no difference between the conventional approach group and the SuperPATH group, concerning the cup inclination angle (MD = − 1.20, 95% CI − 2.79 to 0.40, *I*^2^ = 0%, *p* = 0.10, Fig. 9).

#### 4. Postoperative complications

Data on 407 patients (including 186 patients with SuperPATH approach and 221 patients with conventional approaches) were pooled from 6 RTCs analyzing the postoperative complications. Compared with conventional approach group, the frequency of risk for postoperative complications was lower in the SuperPATH group (OR = 0.83, 95% CI 0.12 to 5.98, *I*^2^ = 37%, *p* = 0.81, Fig. 10).

### THA-subgroup analysis

The comparison of the results between the overall meta-analysis for THA/HA and the THA-subgroup is shown in Table 3. The results of the THA-subgroup did not differ from the overall effect in a clinically relevant manner.

**Table 3:** Comparison of the results between the overall meta-analysis for THA/HA and the THA-subgroup

	THA/HA		THA	
	Pts; RCTs, n	Results	Pts; RCTs, n	Results
**Operation time**	630; 10 [22–27, 29–32]	MD = 18.40, 95% CI 5.38 to 31.42, *I*^2^ = 98%, *p* = 0.01	398; 6 [22–25, 30, 32]	MD = 13.79, 95% CI − 6.47 to 34.05, *I*^2^ = 96%, *p* = 0.14
**Incision length**	552; 8 [22–25, 27, 30–32]	MD = − 4.84, 95% CI − 7.04 to − 2.64, *I*^2^ = 99%, *p* < 0.01	398; 6 [22–25, 30, 32]	MD = − 5.47, 95% CI − 8.34 to − 2.60, *I*^2^ = 99%, *p* < 0.01
**Intraoperative blood loss**	568; 9 [22–27, 29, 30, 32]	MD = − 29.84, 95% CI − 133.69 to 74.02, *I*^2^ = 99%, *p* = 0.53	398; 6 [22–25, 30, 32]	MD = − 20.18, 95% CI − 128.21 to 168.57, *I*^2^ = 99%, *p* = 0.74
**Postoperative drainage volume**	370; 4 [25, 27, 29, 32]	MD = − 75.16, 95% CI − 200.22 to 49.90, *I*^2^ = 100%, *p* = 0.15	238; 2 [25, 32]	MD = − 15.25, 95% CI − 43.11 to 12.61, *I*^2^ = 0%, *p* = 0.09
**Pain VAS 1 day p/o**	222; 4 [22, 24, 30, 32]	MD = − 0.89, 95% CI − 2.96 to 1.19, *I*^2^ = 85%, *p* = 0.27	222; 4 [22, 24, 30, 32]	MD = − 0.89, 95% CI − 2.96 to 1.19, *I*^2^ = 85%, *p* = 0.27
**Pain VAS 3 days p/o**	222;4 [22, 24, 30, 32]	MD = − 1.11, 95% CI − 2.35 to 0.13, *I*^2^ = 75%, *p* = 0.06	222; 4 [22, 24, 30, 32]	MD = − 1.11, 95% CI − 2.35 to 0.13, *I*^2^ = 75%, *p* = 0.06
**Pain VAS 7 days p/o**	348; 4 [23, 30–32]	MD = − 1.39, 95% CI − 2.57 to − 0.21, *I*^2^ = 87%, *p* = 0.03	286; 3 [23, 30, 32]	MD = − 1.15, 95% CI − 2.68 to 0.38, *I*^2^ = 86%, *p* = 0.08
**Pain VAS 3 months p/o**	182; 4 [22–24, 31]	MD = − 0.10, 95% CI − 0.85 to 0.65, *I*^2^ = 61%, *p* = 0.70	120; 3; 182; 4 [22–24]	MD = 0.02, 95% CI − 1.37 to 1.42, *I*^2^ = 74%, *p* = 0.95
**Pain VAS 12 months p/o**	120; 3 [22–24]	MD = − 0.09, 95% CI − 0.32 to 0.13, *I*^2^ = 0%, *p* = 0.22	120; 3 [22–24]	MD = − 0.09, 95% CI − 0.32 to 0.13, *I*^2^ = 0%, *p* = 0.22
**Hospitalization period**	242; 4 [22, 23, 27, 28]	MD = − 2.80, 95% CI − 6.61 to 1.02, *I*^2^ = 96%, *p* = 0.10	150; 3 [22, 23, 28]	MD = − 1.97, 95% CI − 7.49 to 3.56, *I*^2^ = 93%, *p* = 0.27
**HHS 7 days p/o**	246; 4 [23, 26, 28, 31]	MD = 10.24, 95% CI 0.27 to 20.21, *I*^2^ = 96%, *p* = 0.05	146; 2 [23, 28]	MD = 5.40, 95% CI − 5.82 to 16.62, *I*^2^ = 7%, *p* = 0.10
**HHS 14 days p/o**	112; 3 [22–24]	MD = − 1.88, 95% CI − 24.88 to 21.13, *I*^2^ = 73%, *p* = 0.76	112; 3 [22–24]	MD = − 1.88, 95% CI − 24.88 to 21.13, *I*^2^ = 73%, *p* = 0.76
**HHS 1 month p/o**	214; 3 [23, 25, 26]	MD = 4.49, 95% CI − 2.96 to 11.94, *I*^2^ = 93%, *p* = 0.12	176; 2 [23, 25]	MD = 3.12, 95% CI − 14.84 to 21.08, *I*^2^ = 94%, *p* = 0.27
**HHS 3 months p/o**	516; 8 [22–25, 28, 31–33]	MD = 1.64, 95% CI − 2.91 to 6.19, *I*^2^ = 95%, *p* = 0.42	454; 7 [22–25, 28, 32, 33]	MD = 1.02, 95% CI − 4.18 to 6.23, *I*^2^ = 95%, *p* = 0.65
**HHS 6 months p/o**	382; 6 [22, 24, 25, 28, 31, 32]	MD = 0.63, 95% CI 0.11 to 1.15, *I*^2^ = 0%, *p* = 0.03	320; 5 [22, 24, 25, 28, 32]	MD = 0.65, 95% CI − 0.07 to 1.36, *I*^2^ = 0%, *p* = 0.07
**HHS 12 months p/o**	174; 4 [22–24, 28]	MD = 0.64, 95% CI − 0.36 to 1.64, *I*^2^ = 0%, *p* = 0.13	174; 4 [22–24, 28]	MD = 0.64, 95% CI − 0.36 to 1.64, *I*^2^ = 0%, *p* = 0.13
**Cup anteversion angle**	160; 4 [22–24, 30]	MD = − 0.62, 95% CI − 1.51 to 0.28, *I*^2^ = 11%, *p* = 0.12	160; 4 [22–24, 30]	MD = − 0.62, 95% CI − 1.51 to 0.28, *I*^2^ = 11%, *p* = 0.12
**Cup abduction angle**	160; 4 [22–24, 30]	MD = − 1.20, 95% CI − 2.79 to 0.40, *I*^2^ = 0%, *p* = 0.10	160; 4 [22–24, 30]	MD = − 1.20, 95% CI − 2.79 to 0.40, *I*^2^ = 0%, *p* = 0.10
**Postoperative complications**	407; 6 [24, 26, 27, 29, 30, 32]	OR = 0.83, 95% CI 0.12 to 5.98, *I*^2^ = 37%, *p* = 0.81	237; 3	OR = 2.37, 95% CI 0.03 to 206.84, *I*^2^ = 20%, *p* = 0.49

*THA* total hip arthroplasty, *HA* hemiarthroplasty, *Pts* patients

## Discussion

### Main and new findings

Twelve randomized controlled trials with 726 patients were included in this meta-analysis. The SuperPATH approach group consisted of 346 patients; the conventional approach group consisted of 380 patients. In general, our meta-analysis indicated that hip replacement via SuperPATH approach was superior to hip replacement via conventional approaches regarding the investigated outcomes. SuperPATH approach for hip replacement showed better results on decreasing incision length and early postoperative pain intensity. SuperPATH approach for hip replacement had a positive influence on short-term postoperative functional outcome. The two approaches for hip replacement did not differ in acetabular cup positioning angles, intra- and postoperative blood loss, hospitalization period, and postoperative complications. Hip replacement via SuperPATH approach had a longer operation time than hip replacement via conventional approaches. The results of the THA-subgroup did not differ from the overall effect in a clinically relevant manner. One study out of twelve was a blinded RCT with a level I evidence [41], the other 11 studies were non-blinded RCTs with level II evidence [42–52].

The value of this meta-analysis results from the limitation of inclusion criteria to RCTs and employment of high-quality statistical methods. It is the first meta-analysis comparing the SuperPATH approach with conventional approaches to the hip joint in English language. Another advantage is that we considered differences between studies on hip replacement with THA and HA.

#### Operation time

The operation time was 18.4 min longer in hip replacement via SuperPATH approach compared to hip replacement via conventional approaches. In a 2018 Chinese meta-analysis by Li, the operation time was 12.5 min longer in hip replacement via SuperPATH approach compared to hip replacement via conventional approaches [59]. The other Chinese meta-analysis by Sun showed indifferent operation times for both groups [60]. A 2015 study by Rasuli showed that there is a persistent learning curve for surgeons using SuperPATH approach in hip replacement surgery [78]. This finding might be an explanation for longer operation time, since SuperPATH is a relatively new approach. Furthermore, we compared the results with a 2013 meta-analysis with 1174 included patients on hip replacement via mini-incision approaches versus hip replacement via conventional approaches by Xu et al. [36]. The operation time between the two groups was indifferent.

#### Incision length

The incision length in hip replacement via SuperPATH approach was 4.8 cm shorter compared to hip replacement via conventional approaches. In the two Chinese meta-analyses, the incision length was even shorter with 5.7 [59] and 7.5 cm [60]. A 2013 meta-analysis by Moskal with 3548 included hip replacements showed that short-term recovery favors limited incision approaches over standard incision approaches in THA [79].

#### Intraoperative blood loss and postoperative drainage volume

The intraoperative blood loss and the postoperative drainage volume in hip replacement via SuperPATH approach showed no difference compared to hip replacement via conventional approaches. The 2018 meta-analysis by Sun also showed equivalent results for the intraoperative blood loss [60]. The intraoperative blood loss in the meta-analysis by Li was almost indifferent with 2.2 ml [59] less in hip replacement via SuperPATH approach compared to hip replacement via conventional approaches. Furthermore, we compared the results with the meta-analysis by Xu on hip replacement via mini-incision approaches versus hip replacement via conventional approaches [36]. They found better results in hip replacement via mini-incision approaches with 111.5 ml less intraoperative blood loss compared to hip replacement via conventional approaches. The meta-analysis by Li found better results in hip replacement via SuperPATH approach with 98.4 ml less postoperative drainage volume compared to hip replacement via conventional approaches [59].

#### Pain VAS

The early pain VAS in hip replacement via SuperPATH approach was 1.4 points lower 7 days postoperatively compared to hip replacement via conventional approaches. The meta-analysis by Li came to similar results for early pain VAS. They found that the early pain VAS in hip replacement via SuperPATH approach was 1.6 points lower 1 day postoperatively, 1.7 points lower 3 days postoperatively and 1 point lower 7 days postoperatively [59]. The 2013 meta-analysis by Xu did not show differences in postoperative administration of pain medication between hip replacement via mini-incision and conventional approaches [36]. In our meta-analysis, we did not find differences in pain VAS between hip replacements via SuperPATH and conventional approaches 1, 3 days, 3 and 12 months postoperatively.

#### Hospitalization period

The hospitalization period in hip replacement via SuperPATH approach was indifferent compared to hip replacement via conventional approaches. The 2018 meta-analysis by Sun found a 2–3 days shorter hospitalization period in hip replacement via SuperPATH approach. The 2013 meta-analysis by Xu showed that the hospitalization period was 0–1 days shorter in hip replacements via mini-incision compared to hip replacements via conventional approaches.

#### HHS

The early HHS at 7 days postoperatively was 10.2 higher in hip replacement via SuperPATH approach compared to hip replacement via conventional approaches. The subsequent HHS at 14 days, 1, 3, 6 and 12 months postoperatively were almost equal in both groups. The 2018 meta-analysis by Sun showed no difference in both groups [60]. The other Chinese meta-analysis by Li, however, found better results in hip replacement via SuperPATH approach with 4.3 more points compared to hip replacement via conventional approaches at 3 months postoperatively. There was no difference between the two approaches 1 and 6 months postoperatively [59]. The 2013 meta-analysis by Xu did not show differences in HHS between hip replacement via mini-incision and conventional approaches [36].

#### Acetabular cup positioning angle

Our meta-analysis did not find any differences in the acetabular cup positioning angles, neither in anteversion nor in inclination, in hip replacement via SuperPATH approach compared with conventional approaches. The meta-analyses by Li, Sun, and Xu came to the same results, comparing hip replacement via SuperPATH approach respectively mini-incision approach with hip replacements via conventional approaches [36, 59, 60].

#### Postoperative complications

Our meta-analysis and the meta-analysis by Li showed slightly better results for postoperative complications in hip replacement via SuperPATH approach compared to conventional approaches [59].

### Limitations

The limitations to this meta-analysis are as follows: First, the long-term outcomes of SuperPATH approach were not considered. Second, SuperPATH is a relatively new approach to the hip joint with a necessary learning curve, which might influence the operation time in disadvantage for SuperPATH approach. Third, this meta-analysis did not consider the possible influence of the operating surgeons, the usage of bone cement or the types of implants used for hip replacement. Fourth, in some cases of the investigated outcomes, the included studies were too heterogeneous to be comparable. This may lead to questionable meta-analytical results. Lastly, only one study reported sufficient blinding, which might affect the final outcomes.

## Conclusions

Our overall findings suggested that the short-term outcomes of SuperPATH approach in hip replacement were better compared to conventional approaches. SuperPATH approach showed better results in decreasing incision length and early pain intensity as well as improvement of short-term functional outcome. Long-term outcomes of SuperPATH approach need to be investigated.

## Supplementary information

**Additional file 1.** Search strategy

## Data Availability

The data are available from the corresponding author upon reasonable request.

## Abbreviations

CNKI: Chinese National Knowledge Infrastructure
HA: Hemiarthroplasty
HHS: Harris Hip Score
RCT: Randomized controlled trial
SuperPATH: Supercapsular percutaneously assisted approach in total hip arthroplasty
THA: Total hip arthroplasty
VAS: Visual analog scale
