# Supplementary material for: Comparison of short-term outcomes between SuperPATH approach and conventional approaches in hip replacement: a systematic review and meta-analysis of randomized controlled trials
Source: J Orthop Surg Res. 2020 Sep 17;15:420. doi: 10.1186/s13018-020-01884-3 (PMC7499876; doi:10.1186/s13018-020-01884-3)
Supplement: Supplementary file 1 — Additional file 1. Search strategy [file 13018_2020_1884_MOESM1_ESM.docx]

Appendix :

I. Search strategy **PubMed:**

((SuperPATH) OR (Supercapsular Percutaneously-Assisted Total Hip)) ti,ab.

II. Search strategy **CNKI**:

(SuperPATH) OR (Supercapsular Percutaneously-Assisted Total Hip) in Title

III. Search strategy **Cochrane Library**:

((SuperPATH) OR (Supercapsular Percutaneously-Assisted Total Hip)) in Title Abstract Keyword

IV. Search Strategy **Google Scholar**:

SuperPATH

V. Search strategy **Clinical Trials:**

(SuperPATH) OR (Supercapsular Percutaneously-Assisted Total Hip)
